# Allergen Labeling for Eggs as Ingredients of Pre-packaged Foods (Food-allergy)

**DOI:** 10.14252/foodsafetyfscj.D-21-00028

**Published:** 2021-12-24

**Authors:** Food Safety Commission of Japan

## Abstract

Basic Act on allergic diseases measures was enforced in 2015, in order to improve the living environment of Japanese population via enhancing food labeling of concerned allergic ingredients. Food Safety Commission of Japan (FSCJ), then, deemed it necessary to examine this Japanese allergen labeling system. Labeling is currently mandatory for 7 ingredients, and in addition, recommended for 21 distinct ingredients. FSCJ chose to conduct a self-tasking risk assessment on hen-eggs labeling, as hen-eggs show high prevalence of allergy cases out of those presented ingredients. Hen-eggs were specifically focused on this assessment due to the available amount of data. There have been no incidents of hen-egg protein-derived allergic reactions at levels below the “threshold concentration”, which was set at 10 μg of allergenic protein per 1 g of food for the labeling purpose of Japan’s system. On the practical aspect, allergen contamination in pre-packaged foods is continuously prevented through good hygiene practices. Introduction of mandatory HACCP-based approach in the food industry of Japan contributed to appropriate controls, including prevention of labeling errors among others. In conclusion, FSCJ judged the current allergen labeling system on hen-eggs in Japan to be generally appropriate based on the currently available evidences.

## Conclusion in Brief

Allergen labeling on pre-packaged foods was introduced in 2001, prior to the establishment of the Food Safety Commission of Japan (FSCJ). In 2015, Basic Act on Allergic Diseases Measures was enforced. It was established in Article 15 that the national government must take measures to improve the living environment such as reduction and prevention of the air pollution, appropriate management of the forest, enhancement of food labeling concerning allergic ingredients, advancement of the structure of houses and buildings, to prevent aggravation of the allergic diseases and to relieve the symptoms of the patients. FSCJ deemed it necessary to examine Japan’s allergen labeling system^1^^)^ to ensure the food safety of people with food allergies. FSCJ thus decided to conduct the “self-tasking” risk assessment^2^^)^ on allergen labeling on pre-packaged foods.

Food allergies are caused by the ingestion of even tiny amounts of food allergens. To prevent food allergies of consumers, labeling of specified ingredients in pre-packaged food should be effective, regardless of the intention of manufacturer’s use.

Seven ingredients are mandated to be on the labeling and the other 21 ingredients are recommended to be on the labeling in Japan^3^^)^. FSCJ chose poultry eggs for its self-tasking risk assessment due to the high prevalence of egg allergy among food allergies, and the abundance of scientific findings on egg allergy in relative to other allergic ingredients.

The specific ingredient of edible poultry eggs are termed “eggs” in general, but most of the scientific findings obtained were limited to hen-eggs. Hen-eggs are the most consumed poultry eggs in Japan. FSCJ focused mainly the scientific findings related to hen-egg allergy, although available data are not ample as the scientific information.

Medical doctors often advise patients with hen-egg allergy to check allergen labeling before consuming pre-packaged foods. Reportedly, most patients with food allergies actually examine labels at their purchase of pre-packaged foods. These patients are, thus, assumed to keep away from foods labeled with “eggs” in principle. Clinicians do not see problems with the current allergen labeling system as far as the patients avoid eating pre-packaged foods labeled with “eggs”, except the cases from the defected labeling. In fact, there is no case reports are available currently to the best knowledge; there have been no incidents of allergic reactions induced by hen-egg protein at levels below the “threshold concentration”. The threshold concentration is 10 μg of allergenic protein per 1 gram of food for the purpose in Japan’s allergen labeling system.

As for the labeling of “eggs” on pre-packaged foods, domestic inspection reports suggested that concentrations of the “eggs” protein in pre-packaged foods without “eggs” labeling were kept at sufficiently low levels^4^^)^. The number of orders and instructions based on the Food Labeling Act are limited. Only a few voluntary recall cases due to inappropriate labeling are annually reported. These include the incidents of “eggs” detection as an ingredient in food products, unexpectedly containing “eggs”, and also defective labeling on food products necessary to indicate “eggs”.

In practical aspects, allergen management to reduce risks has been ensured by good hygiene practices and standard operational procedures in the food industry of Japan. Allergen contamination in pre-packaged foods has been prevented through continual good hygiene practices (minimizing the likelihood of allergen cross-contact, and appropriate cleaning and washing in the facilities).

Planning and implementation of standard operational procedures in the process of labeling of food products are also important to avoid erroneous labeling. With mandatory introduction of Hazard-Analysis and Critical-Control-Point (HACCP)-based approach in the food industry of Japan, appropriate control measures including prevention of labeling errors will be well implemented, and consequently quality-control and quality-assurance for appropriate allergen labeling will be further promoted.

The amount of allergens yielding allergic symptoms may vary depending on individuals. A few micrograms of hen-egg protein may result in the allergic symptoms for some individuals. All patients with food allergies including hen-egg allergy are thus recommended to follow the doctors’ guidance, prior to consume specific pre-packaged foods. Occurrence of allergic reactions induced by consumption of pre-packaged foods are extremely low among patients with hen-egg allergy. FSCJ judged the current allergen labeling system on “eggs” in Japan to be generally appropriate, given the evidence available.

Continual accumulations of scientific evidence including secular trends in the prevalence of food allergies based on nation-wide epidemiological surveys and research data, and data on eliciting doses determined by oral food challenges are necessary for the successive refinement of risk assessment of food allergens.
